# Investigating the Physical Adsorption of DCPD/Furfural and H_2_ Adsorption–Dissociation Behaviors in RE-MOFs

**DOI:** 10.3390/molecules30091954

**Published:** 2025-04-28

**Authors:** Muye Niu, Zuoshuai Xi, Chenhui He, Wenting Ding, Shanshan Cheng, Juntao Zhang, Hongyi Gao

**Affiliations:** 1 Beijing Key Laboratory of Function Materials for Molecule & Structure Construction, School of Materials Science and Engineering, University of Science and Technology Beijing, Beijing 100083, China; niumuye913@163.com (M.N.); xi_zuoshuai@163.com (Z.X.); d202410306@xs.ustb.edu.cn (W.D.); css7654321@126.com (S.C.); zjtcandy@163.com (J.Z.); 2 Shunde Innovation School, University of Science and Technology Beijing, Foshan 528399, China

**Keywords:** metal–organic framework, adsorption kinetics, hydrogenation, molecular dynamics simulation, DFT

## Abstract

Metal–organic frameworks (MOFs) have emerged as promising catalysts in the hydrogenation of bicyclopentadiene (DCPD) and furfural. The physical adsorption behaviors of substrate molecules and H_2_ within the pore structures of MOFs significantly influence the efficacy of subsequent catalytic reactions. This study employs molecular dynamics (MD) simulations to identify the optimal temperature and pressure conditions for the adsorption of DCPD and H_2_, as well as furfural and H_2_, within rare-earth-element-based MOFs (RE-MOFs). By analyzing the physical adsorption characteristics of 1538 RE-MOFs, we investigate the correlation between pore structures and adsorption capabilities. This exploration has led to the identification of 10 RE-MOF structures that demonstrate superior physical adsorption performance for both DCPD and furfural. Following this initial evaluation, density functional theory (DFT) calculations were conducted to determine the chemisorption energies of H_2_ molecules on these 10 selected RE-MOF structures. Notably, the structure identified as “JALLEQ_clean” exhibited the most optimal overall adsorption performance. This study elucidates the quantitative relationship between the pore structure of RE-MOFs and their physical adsorption performance, clarifying the influence of porosity parameters on adsorption capacity and highlighting the advantages of cluster-type structures in mass transfer and adsorption. The findings provide theoretical guidance for developing high-performance RE-MOF catalysts and offer new insights for the rational design of MOF-based catalytic materials.

## 1. Introduction

DCPD is a pivotal industrial chemical intermediate produced during the high-temperature cracking of petroleum fractions for ethylene and propylene production. It serves as a key precursor in the synthesis of adamantane and its derivatives, insecticides, ethylene-propylene-diene monomer rubber, hydraulic fluids, rubber antioxidants, and pharmaceuticals. Recent research has focused on the catalytic hydrogenation of DCPD to yield tetrahydrodicyclopentadiene (THDCPD), which is utilized as an intermediate in the manufacture of high-energy rocket propellant fuels [1,2,3,4]. Similarly, furfural, an unsaturated heterocyclic aldehyde akin in size to DCPD, can be hydrogenated to produce valuable chemicals such as furfuryl alcohol (FA), 2-methylfuran (2-MF), and cyclopentanone (CPON), which find extensive applications across various industrial sectors [5,6]. Noble metal catalysts, including platinum (Pt), palladium (Pd), and ruthenium (Ru), have demonstrated high activity in the catalytic hydrogenation of both DCPD and furfural [7]. However, their widespread use is limited by high costs and scarcity. Bimetallic catalysts, supported non-noble metal catalysts, and MOFs catalysts can serve as ideal alternatives to high-cost noble metal catalysts [8,9]. Through strategies such as carrier optimization, nanostructure design, and additive incorporation, these catalysts significantly enhance catalytic activity and stability, providing more efficient and economical catalyst options for the catalytic hydrogenation of DCPD and furfural [10].

MOFs are a class of highly ordered, porous crystalline materials formed by the coordination of metal ions or clusters with organic ligands. Their inherent properties, such as low density, large specific surface area, high porosity, and excellent thermal stability, make them highly versatile for applications in gas storage, separation, catalysis, and sensing [10,11,12,13,14,15,16]. Recently, MOFs have showcased exceptional catalytic performance as heterogeneous catalysts in the hydrogenation reactions of DCPD and furfural [17,18,19,20]. For instance, Fu et al. synthesized a series of defect-structured MOF-808, achieving selective hydrogenation of furfural to furfuryl alcohol with a conversion rate of 99% and 94.4% selectivity under conditions of 263 K and a reaction time of two hours. Zhou et al. developed cerium-based MOFs employing a modulation-induced defect engineering strategy, identifying Ce-MOF-801-50eq as the most effective catalyst, achieving a full conversion rate of DCPD [21]. Such studies provide crucial insights for the rational design of RE-MOFs intended for the catalytic hydrogenation of DCPD.

MOFs, characterized by their porous structures and surface-rich active sites, serve as promising catalytic materials. They facilitate the initial physical adsorption of substrate molecules onto the catalyst surface through weak interactions such as van der Waals forces and electrostatic interactions. This process is followed by chemical adsorption, where substrate molecules form chemical bonds with the catalyst surface, often accompanied by electron transfer [22,23]. Typically, physical adsorption occurs prior to chemical adsorption, allowing molecules to approach the surface via weak interactions. This enhances the likelihood of contact with active sites, thereby creating favorable conditions for chemical adsorption. Furthermore, physical adsorption extends the residence time of substrate molecules on the catalyst surface, increasing the local concentration of reactants [24]. This heightened concentration elevates the frequency of collisions between molecules and active sites, thereby improving the likelihood and rate of chemical adsorption. Consequently, the physical adsorption capacity of MOFs for substrate molecules significantly influences the kinetics of chemical adsorption and, ultimately, the catalytic activity of reactions. It is essential to explore the factors that affect the physical adsorption performance of MOFs, which will aid in the design and screening of appropriate frameworks.

In this study, we employed molecular dynamics simulations to ascertain the optimal temperature and pressure conditions for the adsorption of DCPD and hydrogen H_2_, as well as furfural and H_2_, in RE-MOFs. Through an analysis of physical adsorption results from 1538 RE-MOFs, we investigated the relationship between the pore structure of these frameworks and their capacity for physical adsorption. Subsequently, density functional theory (DFT) calculations were performed to determine the chemical adsorption energies of hydrogen for the top 10 RE-MOFs exhibiting the highest adsorption capacities for DCPD and furfural. Ultimately, the results demonstrated that the “JALLEQ_clean” framework exhibited the optimal comprehensive performance in both the physical adsorption of furfural and DCPD, and the chemical adsorption of H_2_.

## 2. Results and Discussions

**Figure 1:**
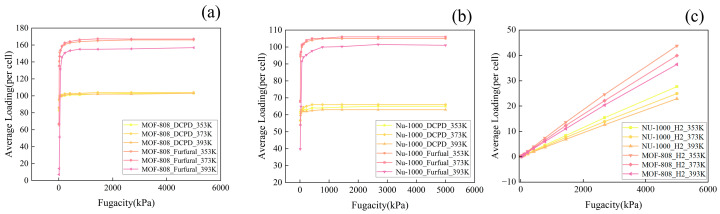
(**a**,**b**) display the adsorption isotherms for the physical adsorption of DCPD and furfural molecules on MOF-808 and NU-1000, respectively; (**c**) illustrates the adsorption isotherms for the physical adsorption of H_2_ molecules on MOF-808 and NU-1000.

To identify the optimal temperature and pressure conditions for the physical adsorption of DCPD, furfural, and hydrogen H_2_ in RE-MOFs, molecular dynamics simulations were conducted. Dual-component adsorption isotherms of DCPD, furfural, and H_2_ on MOF-808 and NU-1000 were obtained (Figure 1). Both NU-1000 and MOF-808 are renowned for their exceptional performance in the catalytic hydrogenation of olefins, alkynes, unsaturated aldehydes, and CO_2_, characterized by their high specific surface areas, porosity, and thermal stability [25].

Figure 1a depicts the adsorption isotherms of DCPD and furfural on MOF-808 at three distinct temperatures: 353 K, 373 K, and 393 K. Notably, the adsorption capacity of furfural consistently exceeds that of DCPD across all examined temperatures. Within the pressure range of 0–500 kPa, the adsorption capacity increases rapidly with escalating pressure. However, beyond 500 kPa, the rate of adsorption growth diminishes, stabilizing at 2000 kPa. At 373 K and 353 K, the furfural adsorption capacities are comparable, averaging 160 molecules per unit cell at 2000 kPa. Conversely, at 393 K, the adsorption capacity for furfural is reduced compared to 373 K and 353 K, suggesting that furfural molecules experience more vigorous motion within the MOF channels at elevated temperatures. This increase in thermal energy facilitates the detachment of adsorbed furfural molecules from their sites.

In contrast, the adsorption of DCPD on MOF-808 remains relatively consistent across the three temperatures, with an average of approximately 100 DCPD molecules per unit cell at 2000 kPa. The corresponding adsorption isotherms for DCPD and furfural on NU-1000 at 353 K, 373 K, and 393 K are illustrated in Figure 1b. Similar to MOF-808, the furfural adsorption capacity on NU-1000 is nearly identical at 373 K and 353 K, with a marked decrease at 393 K. At all temperatures, adsorption stabilizes at 2000 kPa, reaching capacities of up to 105 furfural molecules per unit cell at 373 K and 353 K. For DCPD on NU-1000, the adsorption capacity at 2000 kPa decreases in the following order: 373 K, 353 K, and then 393 K.

Figure 1c presents the adsorption isotherms of H_2_ on both NU-1000 and MOF-808. The adsorption capacities of both MOFs for H_2_ increase approximately linearly with pressure, with minor variations observed at different temperatures. The capacity follows the following order: 353 K, 373 K, and then 393 K. This trend can be attributed to the small molecular size of H_2_, as the pore channels in both MOF-808 and NU-1000 are significantly larger than H_2_ molecules. As the temperature increases, the frequency of thermal motion among H_2_ molecules accelerates, enhancing their likelihood of being captured by adsorption sites within the pore channels. However, this phenomenon has a marginal impact on the overall adsorption capacities when the pore size is substantially larger than that of H_2_, and the temperature increment is relatively modest. Based on this comprehensive analysis, the optimal conditions for the physical adsorption of DCPD and furfural are determined to be 373 K and 2000 kPa.

**Figure 2:**
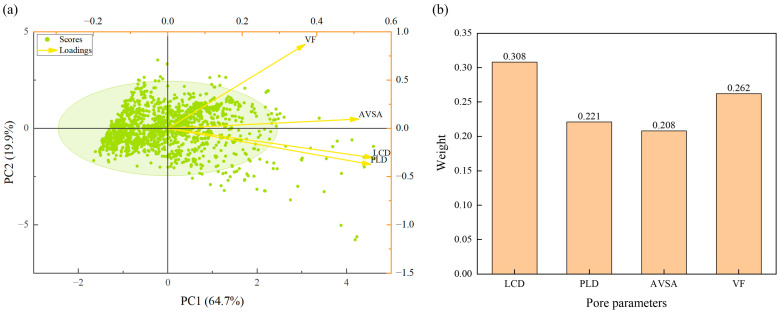
(**a**) Principal Component Analysis; (**b**) Weighting Analysis.

Based on the optimal temperature and pressure conditions derived from adsorption isotherms, we calculated the average physical adsorption capacity per unit cell for DCPD and furfural molecules across 1538 rare-earth (RE)-MOF structures selected from the CoRE database under these specific conditions. To identify the primary factors influencing the physical adsorption capacity, we performed a Principal Component Analysis (PCA) and Weighting Analysis to examine the relationships between adsorption capacity and pore parameters, including PLD, LCD, VF, and AVSA, as illustrated in Figure 2 [26].

The contribution rate and cumulative contribution rate of each principal component were calculated based on eigenvalues. According to the criterion of the cumulative variance contribution exceeding 80%, the first two principal components were extracted. Among them, the first principal component (PC1) accounted for 64.657% of the variance, while the second (PC2) contributed 19.916%. Together, these two principal components collectively explained 84.573% of the original variable information.

Factor loadings were computed to reveal the correlation between principal components and pore structure parameters. LCD, PLD, and VF exhibited strong loadings on PC1 (0.887, 0.877, and 0.825, respectively), indicating a high correlation among these three parameters. Thus, PC1 represents a comprehensive metric of pore size and surface characteristics, serving as the primary driver influencing the adsorption performance of different structures. VF also showed a moderate loading on PC2 (0.593), suggesting that VF is a key factor affecting substrate molecule transport and diffusion efficiency.

Subsequently, the weights of the four pore parameters were determined, ranked as follows: LCD (0.308) > VF (0.262) > PLD (0.221) > AVSA (0.208). This demonstrates that LCD has the most significant influence on the adsorption performance of diverse porous structures.

**Figure 3:**
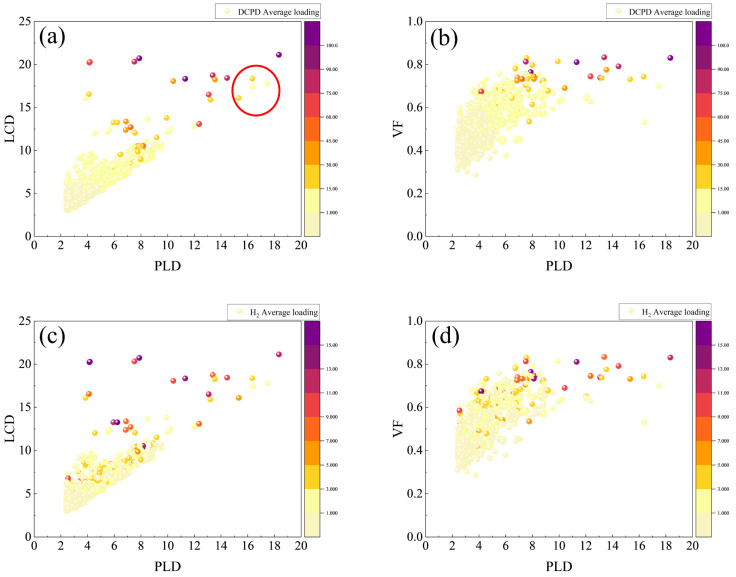
The potential relationships between the adsorption capacities of DCPD and H_2_ and pore parameters such as PLD, LCD, VF, and AVSA are depicted. The *x*-axis in all graphs represents PLD (Å), with the y-axes in (**a**,**c**) representing LCD (Å), and in (**b**,**d**) representing VF. The color intensity indicates the magnitude of adsorption capacity, where (**a**,**b**) correspond to the adsorption capacity of DCPD molecules, and (**c**,**d**) correspond to the adsorption capacity of H_2_ molecules.

Building upon the insights gained from the linear regression analysis, we further investigated the influence patterns of PLD, LCD, and VF on the adsorption capacities of DCPD and H_2_ molecules. Figure 3 presents a comprehensive visualization of these relationships, with the *x*-axis representing PLD and the y-axes denoting LCD and VF. The color intensity of the data points corresponds to the magnitude of the adsorption capacities for both DCPD and H_2_.

Our observations indicate that points with higher adsorption capacities are predominantly situated in regions characterized by larger LCD and VF values. Notably, these points appear to be randomly distributed across various PLD values without a discernible trend; this suggests that when PLD is comparable, the adsorption capacities of DCPD and H_2_ exhibit an increasing trend, with larger LCD and VF values. Specifically, when PLD is less than 0.6 Å, the adsorption capacity for DCPD remains generally low. In contrast, for H_2_, points associated with high adsorption capacities are observed across a broader range of PLD values, indicating that PLD has a relatively minor impact on the adsorption capacity of H_2_.

Further analysis of Figure 3a,c reveals a significant proportion of data points with high adsorption capacities within the LCD range of 15–20 Å. This suggests that RE-MOFs may demonstrate enhanced adsorption performance for both DCPD and H_2_ when LCD values exceed 15 Å. Conversely, Figure 3b,d illustrate that the adsorption performance of RE-MOFs for DCPD is generally poor when porosity is below 0.6; similarly, the H_2_ adsorption performance is suboptimal when porosity falls below 0.5. These findings collectively imply that RE-MOFs may exhibit excellent adsorption capabilities for both DCPD and H_2_ when porosity exceeds 0.6.

**Figure 4:**
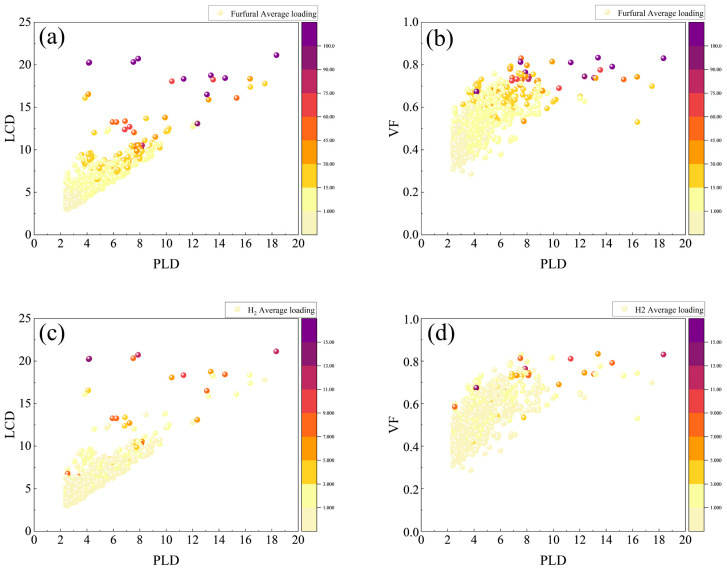
The potential relationships between the adsorption capacities of furfural and H_2_ and pore parameters such as PLD, LCD, VF, and AVSA are depicted. The *x*-axis in all graphs represents PLD (Å), with the *y*-axes in (**a**,**c**) representing LCD (Å), and in (**b**,**d**) representing VF. The color intensity indicates the magnitude of adsorption capacity, where (**a**,**b**) correspond to the adsorption capacity of DCPD molecules, and (**c**,**d**) correspond to the adsorption capacity of H_2_ molecules.

The adsorption capacity of furfural molecules exhibits a pattern analogous to that observed for DCPD, as illustrated in Figure 4. Specifically, the adsorption capacity of furfural increases with both LCD and VF. Notably, a significant proportion of data points reflecting a low DCPD adsorption capacity are identified when the pore length diameter (PLD) is less than 0.5 Å. Conversely, a high concentration of structures exhibiting elevated adsorption capacities is concentrated within the LCD range of 15 to 20 Å. Furthermore, the adsorption capacities of RE-MOFs for both DCPD and H_2_ are generally constrained when the porosity is below 0.6.

Due to the relatively smaller molecular size of furfural compared to DCPD, as well as its simpler spatial structure, the overall adsorption capacity of RE-MOFs for furfural surpasses that for DCPD. This trend is supported by the increased number of structures demonstrating adsorption capacities within the range of 1 to 10 for furfural relative to DCPD. In the context of the linear regression analysis, the Pearson correlation coefficients corresponding to the adsorption of furfural molecules consistently exceed those for DCPD. This observation allows us to infer that the adsorption capacity of smaller substrate molecules within RE-MOFs is more profoundly influenced by pore size, underscoring the significance of pore architecture in optimizing substrate interactions.

It is noteworthy that within the PLD range of 16–18 Å and the LCD range of 15–20 Å, three RE-MOFs exhibit DCPD and furfural adsorption capacities below 15, as marked by red circles in Figure 3a. This observation diverges from the previously established trend indicating that “larger LCD and VF values correspond to higher adsorption capacities for DCPD and H_2_”. To further investigate the influence of MOF pore structures on adsorption capacity, we arranged the 1538 RE-MOFs by PLD in descending order and selected the top 25 structures for an in-depth analysis of their adsorption capacities and corresponding structural models.

**Figure 5:**
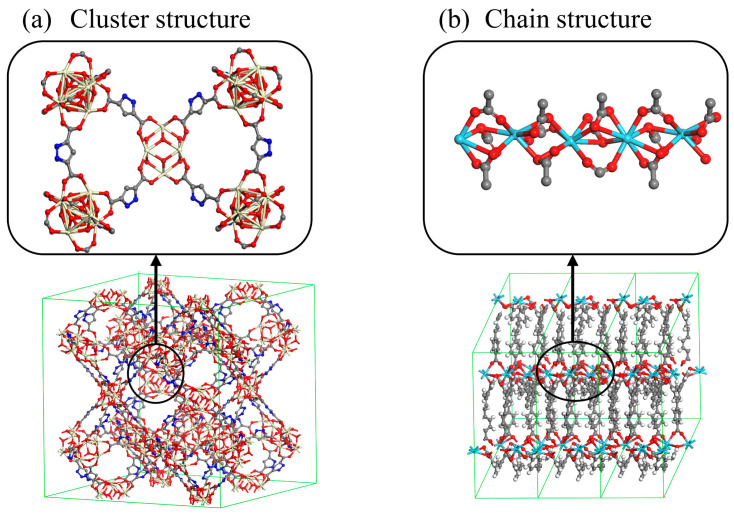
Schematic diagram of cluster-structure SBU and chain-structure SBU.

The analysis revealed that among these 25 RE-MOFs, those with DCPD and furfural adsorption capacities below 15 share a common characteristic: all have SBUs that are chain structures (Figure 5b). In these MOFs, the ligands are densely packed in a direction perpendicular to the pore channels. Despite the large internal pore space, this configuration may impede substrate molecules’ access to the physical adsorption sites. Consequently, even though these structures possess large PLD and LCD values, their physical adsorption capacities remain relatively low.

**Figure 6:**
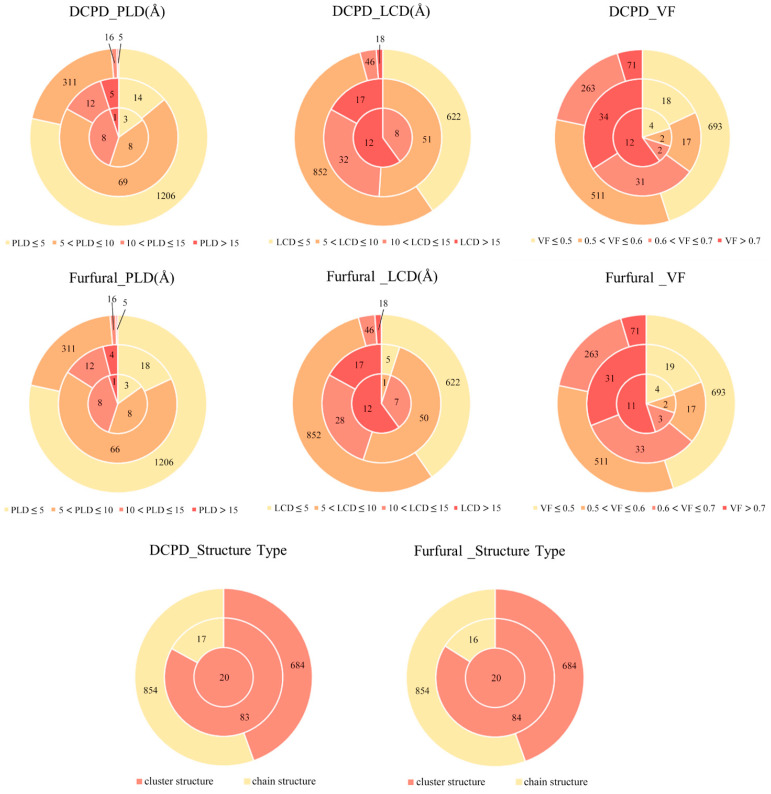
The influence of pore parameters on the physical adsorption of substrate molecules.

The three-layer pie chart in Figure 6, from outer to inner, represents 1538 RE-MOFs, the top 100 RE-MOFs ranked by physical adsorption capacity, and the top 20 RE-MOFs with the highest adsorption capacity, respectively. Notably, structures with PLD > 10 account for only 1.365% (21 structures) in the outermost layer but increase significantly to 45% (9 structures) in the innermost layer, suggesting that larger PLD in the calculated RE-MOFs may correlate with enhanced physical adsorption performance. Furthermore, among the top 20 RE-MOFs, those with LCD > 10 demonstrate exceptionally high proportions (95% for furfural adsorption and 100% for DCPD adsorption analysis). Importantly, all 20 top-performing RE-MOFs in the innermost layer exclusively feature cluster-based SBUs (as illustrated in Figure 5a), clearly indicating that when pore structure dimensions are comparable, RE-MOFs with cluster-type SBUs exhibit superior physical adsorption capabilities compared to their chain-type SBU counterparts.

Furthermore, within the top 20 RE-MOFs ranked by adsorption capacity, the proportion of structures with an LCD exceeding 10 Å is significantly elevated. Remarkably, all 20 RE-MOFs in the innermost layer are characterized by cluster-structure SBUs, as illustrated in Figure 5a. This observation indicates that, when pore structure dimensions are comparable, RE-MOFs with cluster-structure SBUs demonstrate superior physical adsorption performance in comparison to those with chain-structure SBUs. In summary, RE-MOFs featuring cluster-structure SBUs, PLD greater than 10 Å, LCD exceeding 10 Å, and VF greater than 0.6 are most likely to exhibit exceptional physical adsorption performance for DCPD and furfural. Additionally, the physical adsorption performance of RE-MOFs with cluster-structure SBUs may surpass that of RE-MOFs with chain-structure SBUs.

To identify RE-MOFs with optimal catalytic hydrogenation performance for DCPD and furfural, structural defects were intentionally introduced into RE-MOFs demonstrating high physical adsorption capacities. Subsequent DFT calculations were employed to ascertain the hydrogen chemisorption energies, thereby assessing the potential catalytic activity of these modified frameworks for hydrogenation reactions.

Existing research suggests that the introduction of defects can significantly enhance the adsorption properties of MOFs. For example, defects can create new active sites, modify the surface charge, and alter the distribution of functional groups within the frameworks, thus improving interactions with adsorbate molecules and their selectivity. Furthermore, defects may optimize pore size distribution, increase porosity and specific surface area, and enhance the diffusion pathways available to adsorbate molecules, ultimately boosting adsorption rates and efficiencies [27,28,29,30,31,32].

Prior to conducting DFT calculations, we computed the physical adsorption capacities of the top 10 RE-MOFs with introduced defects for both DCPD and furfural molecules. As anticipated, the physical adsorption capacities of the defect-introduced RE-MOFs demonstrated a modest increase compared to their pristine counterparts, with several structures exhibiting similar physical adsorption capacities to those observed prior to defect introduction, as detailed in Table 1.

Table 2 presents the H_2_ chemisorption energies of the top 10 RE-MOFs, selected based on their physical adsorption performance. Notably, the structures “ALULEZ_clean”, “XOFGEG_clean”, “ATEYOP_clean”, “DUFKAS_clean”, and “MUWRIH_clean” did not exhibit significant chemisorption capabilities for H_2_ molecules, while “DUFKAS_clean” and “MUWRIH_clean” demonstrated commendable performance in the physical adsorption of DCPD and furfural. Among all evaluated structures, “JALLEQ_clean” exhibited the lowest H_2_ chemisorption energy of 10.41 kcal/mol. Furthermore, this structure also demonstrated the highest physical adsorption capacity among the 1538 RE-MOFs assessed, capable of adsorbing up to 110 DCPD molecules and 120 furfural molecules per unit cell. A comprehensive analysis of both the physical and chemisorption performance reveals that “JALLEQ_clean” stands out as the most effective candidate among the RE-MOFs analyzed in this study, showcasing exceptional potential for facilitating catalytic hydrogenation. (Relevant structural coordinate information can be found in Table S1).

## 3. Materials and Methods

### 3.1. Dynamics

In this study, all physical adsorption calculations were performed using the molecular dynamics software Material Studio 2020. The MOFs model structure files used were sourced from the CoRE database [33,34]. All frameworks were considered rigid during the simulations [35,36,37]. The Sorption module was utilized to calculate the adsorption isotherms and physical adsorption capacities. The simulation method selected was the Adsorption Isotherm, with the Universal force field chosen for the force field settings. Electrostatic interactions were treated using the Ewald summation method, and the grid spacing was set to 0.2 Å. Adsorbate molecule exchange attempts were accepted with a 40% probability, while conformational isomerization, rotation, and translation moves each had a 20% acceptance rate. The simulations employed a restricted torsion angle of 5° and a displacement amplitude of 1 Å for the translational moves. The adsorption process was executed for 2 × 10^8^ cycles, allowing the energy of the entire adsorption system to converge to a reasonable range, after which the computational data were obtained. The calculations employed the Metropolis algorithm. The temperatures were set to 353 K, 373 K, and 393 K, respectively, while the pressure of the adsorption system was varied from 0 to 5000 kPa.

After obtaining the adsorption isotherms at different temperatures, the Fixed Pressure adsorption method was selected in the Sorption module. The Metropolis algorithm was also applied, and the force field and charge settings were consistent with those used in the Adsorption Isotherm method. The pressure for DCPD and furfural was set to 101.3 kPa, while the pressure for hydrogen was set to 2020 kPa. The temperature was fixed at 373 K, as these temperature and pressure conditions were determined to be optimal based on the adsorption isotherm calculations.

### 3.2. Porosity Parameter

The RE-MOFs structures used in this study were all sourced from the CoRE database. The CoRE database provides partial porosity parameters for some RE-MOFs structures, such as Pore Limiting Diameter (PLD), Largest Cavity Diameter (LCD), Void Fraction (VF), and Accessible Volumetric Surface Area (AVSA). For porosity parameters not provided in the CoRE database, the Zeo++ software (Version 0.3) was employed to calculate them [38].

### 3.3. DFT Study

All DFT calculations were performed using the Gaussian 09, Rev. E01 software package [39]. The geometric optimization and single-point energy calculations of MOF structures were conducted using the PBE0 functional [40]. For metal elements, the pseudopotentials of the Stuttgart group were employed [41], while for main-group elements (C, H, O), the 6-31G(d) basis set was used for geometric optimization, and the higher-accuracy 6-311G(d, p) basis set was selected for energy calculations. All DFT calculations incorporated the Grimme’s D3 dispersion correction to account for weak interactions [42]. The Gaussian convergence criteria were applied as maximum force < 0.00045 a.u., RMS force < 0.0003 a.u., and maximum displacement < 0.0018 a.u. Previous studies have proved that this level of theory is enough to obtain the optimized structures and analyze the vibration of MOFs clusters or intermediates adsorbed on MOFs substrates [43,44,45,46,47,48]. The Shermo software package (Version 2.6) [49] was used to calculate the zero-point correction energy (E_ZPE_) and other correction energies before and after geometric optimization at 373 K, the frequency correction factor was set to 0.95 [50], and the adsorption energy calculation was corrected to ensure its accuracy.

In the calculations, the SBU (Secondary Building Unit) structures were obtained by disconnecting the carboxylate groups of the linking ligands and adding hydrogen atoms at the terminal positions to maintain charge neutrality, thereby extracting them from the periodic MOF structures. To simulate the coordination environment of the ligands in the periodic MOF structures, the terminal carbon atoms were frozen. The formula used to calculate the chemical adsorption energy of H_2_ is as follows:

(1) $$E_{\mathrm{ads}}{=E}_{{\mathrm{MOF}/H}_{2}}{\;\text{-}\;E}_{\mathrm{MOF}}{\;\text{-}\;E}_{H_{2}}$$

where $E_{\mathrm{ads}}$ represents the adsorption energy, $E_{{\mathrm{MOF}/H}_{2}}$ denotes the total energy of the MOF structure after adsorbing an H_2_ molecule, $E_{\mathrm{MOF}}$ represents the energy of the MOF structure before adsorbing an H_2_ molecule, and $E_{H_{2}}$ represents the energy of the H_2_ molecule.

## 4. Conclusions

This study investigates the physical adsorption behaviors of DCPD and furfural in RE-MOFs through molecular dynamics simulations and DFT calculations, further evaluating the chemisorption performance of selected RE-MOFs for H_2_. Our results demonstrate that the pore structure of MOFs significantly influences their physical adsorption performance, with LCD and VF emerging as critical factors determining adsorption capacity. By analyzing the physical adsorption data from 1538 RE-MOFs, we identified 10 structures that exhibit excellent performance in the adsorption of both DCPD and furfural. Further DFT calculations were performed to assess the chemisorption energies of these RE-MOFs for H_2_ molecules, leading to the identification of “JALLEQ_clean” as the best-performing structure. This RE-MOF achieves remarkable physical adsorption capacities, accommodating up to 110 DCPD molecules and 120 furfural molecules per unit cell while also exhibiting the lowest hydrogen chemisorption energy of 2.21 kcal/mol. Additionally, our findings reveal that RE-MOFs featuring a cluster structure as the SBU outperform those with a chain structure under conditions where PLD exceeds 10 Å, LCD exceeds 10 Å, and VF exceeds 0.6. In conclusion, this research provides a significant theoretical foundation and computational guidance for the screening and design of RE-MOFs with superior catalytic hydrogenation performance. These insights lay the groundwork for the future development of efficient and cost-effective catalytic materials.

## Figures and Tables

**Table 1:** Comparison of DCPD and furfural adsorption capacities before and after the introduction of structural defects. N_DCPD_ represents the adsorption capacity of DCPD, N_Furfural_ represents the adsorption capacity of furufural.

Structure Name	Unconstructed Defect		Constructed Defects	
	N_DCPD_	N_Furfural_	N_DCPD_	N_Furfural_
JALLEQ_clean	110.095	173.179	110.658	173.023
DUFKAS_clean	101.137	153.937	102.321	155.248
DOMDAL_clean	78.269	117.318	79.786	122.779
ALULEZ_clean	76.632	111.336	76.651	114.25
ZIJSAO_clean	75.009	113.581	76.514	120.183
XOFGEG_clean	71.445	111.885	71.821	112.063
MUWRIH_clean	71.022	120.601	75.372	117.038
PAQMAY_clean	65.366	105.13	67.17	107.811
ATEYOP_clean	48.657	82.965	50.959	88.445
c5dt03091a_c5dt03091a2_clean	45.216	70.819	45.112	75.106

**Table 2:** The chemisorption energies of H_2_ for the 10 RE-MOFs with the optimal physical adsorption performance. E_ads_ represents the chemisorption energy, N_DCPD_/$N_{H_{2}}$ denotes the adsorption capacities of DCPD and H_2_ as determined by molecular dynamics calculations during co-adsorption, and similarly, N_Furfural_/$N_{H_{2}}$ represents the adsorption capacities of furfural and H_2_.

Structure Name	E_ads_ (kcal/mol)	$N_{\mathrm{DCPD}}/N_{H_{2}}$	$N_{\mathrm{Furfural}}/N_{H_{2}}$
ALULEZ_clean	-	76.6/7.9	111.3/6.1
ATEYOP_clean	-	48.7/11.3	82.9/6.9
c5dt03091a_c5dt03091a2_clean	13.99	45.2/7.5	70.8/5.1
DOMDAL_clean	23.34	78.3/9.9	117.3/7.4
DUFKAS_clean	-	101.1/13.3	153.9/7.4
JALLEQ_clean	10.41	110.1/16.9	173.2/12.5
MUWRIH_clean	-	71.1/12.4	120.6/22.1
PAQMAY_clean	16.29	65.4/11.5	106.0/8.1
XOFGEG_clean	-	71.4/18.5	111.9/13.9
ZIJSAO_clean	49.99	75.0/10.1	113.6/8.3

## Data Availability

No new data were created or analyzed in this study. Data sharing is not applicable to this article.

## Supplementary Materials

The following Supporting Information can be downloaded at: https://www.mdpi.com/article/10.3390/molecules30091954/s1, Table S1. Atomic coordinate data of RE-MOFs clusters. File S1. Calculation result of physical adsorption amount. Text S1. Frequency Analysis.
