# Supplementary material for: Investigating the Physical Adsorption of DCPD/Furfural and H2 Adsorption–Dissociation Behaviors in RE-MOFs
Source: Molecules. 2025 Apr 28;30(9):1954. doi: 10.3390/molecules30091954 (PMC12073282; doi:10.3390/molecules30091954)
Supplement: Supplementary file 1 [file molecules-30-01954-s001.zip › supporting information.pdf]

Supporting Information

Exploring RE-MOFs for catalyzing hydrogenation of bicyclopentadiene and furfural: A MD and DFT study

Muye Niu[1,ab], Zuoshuai Xi[1,a], Chenhui He[ab], Wenting Ding[a], Shanshan Cheng[a], Juntao Zhang[ab], Hongyi Gao[a*]

[a] *Beijing Key Laboratory of Function Materials for Molecule & Structure Construction, School of Materials Science and Engineering, University of Science and Technology Beijing, Beijing 100083, P. R. China.*
[b] *Shunde Innovation School, University of Science and Technology Beijing, Shunde 528399. P. R. China*

E-mail addresses: hygao@ustb.edu.cn.
[1] *These authors contributed equally to this work.*

Table of Contents

1. **Atomic coordinate data of RE-MOFs clusters**

2. **Frequency Analysis**

Table S1. Atomic coordinate data of RE-MOFs clusters

DOMDAL before hydrogenation

	Atom	X	Y	Z
1	O	-1.19393	0.044218	-1.5342
2	C	3.152727	-4.35611	-0.71703
3	O	2.722269	-2.04718	-1.39002
4	O	3.768286	-2.32773	0.511555
5	C	3.234404	-2.83372	-0.52648
6	C	-4.54852	-1.97008	-0.25997
7	C	-5.415	-3.23767	-0.41827
8	O	-3.7806	-1.83784	0.745282
9	O	-4.57016	-1.04854	-1.13998
10	C	-0.16427	-1.03516	1.523255
11	C	-0.66812	-2.32735	2.148268
12	O	-1.02087	-0.14946	1.256528
13	Tb	-2.82587	0.308147	-0.07051
14	O	1.135876	0.752155	-1.18292
15	Tb	2.852665	-0.12662	0.046695
16	O	1.082227	-0.87032	1.32196
17	C	-3.03939	4.621415	0.285702
18	O	-3.06591	2.354842	1.185775
19	O	-3.20584	2.551685	-0.99481
20	C	-3.12236	3.09637	0.148688
21	C	4.603137	2.231277	-0.02088
22	C	5.52831	3.502947	-0.01307
23	O	4.057567	1.818402	-1.0926
24	O	4.378142	1.581918	1.056672
25	H	3.189652	-4.59809	-1.77955
26	H	2.191876	-4.69342	-0.31625

27	H	3.956741	-4.84518	-0.1671
28	H	-4.80705	-4.00899	-0.90071
29	H	-6.27887	-3.02315	-1.04737
30	H	-5.71751	-3.59872	0.565207
31	H	-1.6129	-2.61497	1.681673
32	H	-0.86972	-2.1318	3.206442
33	H	0.07778	-3.11909	2.073208
34	H	-3.43306	5.100725	-0.61031
35	H	-1.98709	4.895269	0.408264
36	H	-3.57964	4.941942	1.177688
37	H	5.590139	3.918757	-1.01756
38	H	6.516562	3.209455	0.347877
39	H	5.111946	4.231193	0.686432
40	H	1.319828	1.463218	-1.80486
41	H	-1.34329	-0.07701	-2.47634
42	H	0.110972	0.421622	-1.32637

DOMDAL after hydrogenation

	Atom	X	Y	Z
1	O	-1.23696	-0.04719	-1.54398
2	C	3.18834	-4.39803	-0.33614
3	O	2.208755	-2.3053	-1.03063
4	O	3.631113	-2.24306	0.648755
5	C	3.000653	-2.90279	-0.24364
6	C	-4.59372	-1.83992	-0.4795
7	C	-5.35351	-3.13548	-0.76983
8	O	-4.06161	-1.65188	0.660773
9	O	-4.45261	-0.94695	-1.37887
10	C	-0.37081	-1.02587	1.64077
11	C	-0.83614	-2.3461	2.21668
12	O	-1.26207	-0.18267	1.305467
13	Tb	-2.93323	0.384603	-0.0533
14	O	3.818043	0.149611	-1.83943
15	Tb	2.586058	-0.20794	0.077587

16	O	0.860183	-0.78702	1.545871
17	C	-2.92332	4.669786	0.267563
18	O	-3.38403	2.422594	1.094349
19	O	-2.89164	2.616342	-1.03482
20	C	-3.08897	3.162314	0.0932
21	C	4.867881	2.304131	-0.03847
22	C	5.61855	3.407122	0.701123
23	O	5.139866	2.136056	-1.24656
24	O	4.004075	1.587657	0.599175
25	H	2.695312	-4.79319	-1.22371
26	H	2.764724	-4.86169	0.559825
27	H	4.25544	-4.63162	-0.34947
28	H	-4.62548	-3.89922	-1.06056
29	H	-6.05485	-2.98609	-1.59066
30	H	-5.86344	-3.47294	0.133186
31	H	-1.78559	-2.64061	1.765589
32	H	-1.00448	-2.20849	3.290055
33	H	-0.07289	-3.11304	2.082734
34	H	-3.08743	5.17547	-0.68387
35	H	-1.89738	4.861071	0.597244
36	H	-3.60498	5.035112	1.03655
37	H	6.205449	3.989679	-0.00696
38	H	6.277913	2.951115	1.445792
39	H	4.912789	4.045313	1.238801
40	H	4.41282	1.008396	-1.7282
41	H	-1.31663	0.063052	-2.49731
42	H	3.610201	-0.04413	-2.75491
43	H	-0.21209	0.182872	-1.30318
44	H	0.917956	0.4991	-1.0814

JALLEQ before hydrogenation

	Atom	X	Y	Z
1	C	-3.06525	-3.57935	-0.79692
2	C	-4.04144	-4.72441	-0.92482
3	O	-3.49199	-2.41177	-0.89236
4	O	-1.84137	-3.91068	-0.59696
5	C	2.337156	0.029233	3.827601
6	C	3.104917	0.039603	5.128269

7	O	2.991201	0.049415	2.754217
8	O	1.068428	0.000225	3.921244
9	C	-3.18083	3.541462	-0.71951
10	C	-4.16279	4.688691	-0.78862
11	O	-3.5932	2.37688	-0.86892
12	O	-1.95788	3.878286	-0.50739
13	C	3.812351	0.071581	-2.78724
14	C	5.062743	0.099882	-3.63456
15	O	3.938939	0.065439	-1.54044
16	O	2.704184	0.055864	-3.42188
17	C	2.990627	3.436299	0.476562
18	C	3.968143	4.563437	0.713283
19	O	3.411316	2.257269	0.576273
20	O	1.796454	3.777924	0.194101
21	O	-4.08689	0.14726	-2.2611
22	C	3.122631	-3.33929	0.497018
23	C	4.144532	-4.4255	0.737301
24	O	3.49785	-2.14411	0.578938
25	O	1.940108	-3.73031	0.227079
26	C	-3.90406	-0.05637	2.486982
27	C	-5.13471	-0.06203	3.362245
28	O	-4.05854	-0.07272	1.243242
29	O	-2.78208	-0.0353	3.094685
30	Ce	-0.03366	-2.49935	-0.20538
31	Ce	-0.1375	2.473848	-0.2116
32	Ce	0.499858	0.000853	-2.56137
33	Ce	-0.59092	-0.0163	2.221074
34	Ce	-2.70662	-0.08385	-0.75251
35	Ce	2.483777	0.039221	0.407783
36	O	-1.53086	-1.38172	0.820965
37	O	0.934051	-1.292	1.347053
38	O	-1.02626	1.300439	-1.86232
39	O	1.428869	1.298513	-1.19274
40	O	0.87114	1.327099	1.361975
41	O	-0.99564	-1.33865	-1.83315
42	O	1.475164	-1.28493	-1.21144
43	O	-1.58675	1.26889	0.785391
44	H	-4.01233	-5.33145	-0.01405
45	H	-3.73531	-5.37244	-1.7525
46	H	-5.05338	-4.35532	-1.09558

47	H	4.180614	0.06662	4.950112
48	H	2.845062	-0.85107	5.709453
49	H	2.802509	0.909113	5.720755
50	H	-5.17554	4.327332	-0.97278
51	H	-3.8622	5.380867	-1.58193
52	H	-4.13386	5.248358	0.151994
53	H	5.077326	-0.77632	-4.29086
54	H	5.957897	0.112039	-3.01156
55	H	5.0453	0.984605	-4.27927
56	H	4.971732	4.180248	0.902059
57	H	3.631537	5.157612	1.569369
58	H	3.976518	5.228678	-0.15581
59	H	4.191434	-5.08138	-0.13786
60	H	3.824303	-5.04192	1.583686
61	H	5.128852	-4.0013	0.939291
62	H	-5.10617	-0.93451	4.023006
63	H	-6.04361	-0.08095	2.759721
64	H	-5.12892	0.825998	4.002603
65	H	-4.42209	1.058742	-2.30148

JALLEQ after hydrogenation

	Atom	X	Y	Z
1	C	-3.16643	-3.44526	-0.83532
2	C	-4.13799	-4.57306	-1.09714
3	O	-3.59837	-2.26678	-0.87697
4	O	-1.96254	-3.79015	-0.60107
5	C	2.443705	-0.01053	3.793442
6	C	3.252961	-0.01313	5.068401
7	O	3.056011	0.010419	2.699543
8	O	1.175112	-0.03048	3.932855
9	C	-3.24867	3.438549	-0.76054
10	C	-4.22366	4.566678	-1.00797
11	O	-3.66986	2.256153	-0.82836
12	O	-2.04884	3.781113	-0.51345
13	C	3.82441	0.076755	-2.75444
14	C	5.074731	0.106216	-3.59981
15	O	3.958803	0.064713	-1.50061
16	O	2.718962	0.067944	-3.37131
17	C	3.003183	3.430168	0.517875

18	C	3.990806	4.54719	0.759509
19	O	3.416129	2.248045	0.581344
20	O	1.804955	3.787187	0.261353
21	O	-4.83621	0.114917	-1.71732
22	C	3.10349	-3.36803	0.456912
23	C	4.124345	-4.4593	0.677027
24	O	3.481214	-2.17546	0.540677
25	O	1.915893	-3.75594	0.195361
26	C	-3.85174	-0.07425	2.562454
27	C	-5.06465	-0.08956	3.46373
28	O	-4.03686	-0.06858	1.318739
29	O	-2.721	-0.06915	3.145116
30	Ce	-0.02065	-2.48634	-0.24682
31	Ce	-0.09658	2.472207	-0.19896
32	Ce	0.454415	0.026604	-2.68211
33	Ce	-0.50614	-0.03474	2.279307
34	Ce	-2.55894	-0.04746	-0.60525
35	Ce	2.484963	0.024342	0.347451
36	O	-1.5364	-1.34235	0.847536
37	O	0.934588	-1.3163	1.349201
38	O	-1.11626	1.299078	-1.73915
39	O	1.403032	1.346334	-1.19523
40	O	0.894913	1.304969	1.377546
41	O	-1.07445	-1.30606	-1.76096
42	O	1.443451	-1.30139	-1.22378
43	O	-1.57571	1.264617	0.868511
44	H	-4.0598	-5.32116	-0.30275
45	H	-3.86349	-5.06823	-2.03493
46	H	-5.16229	-4.2045	-1.1693
47	H	4.32221	0.007781	4.854425
48	H	3.00655	-0.90571	5.652556
49	H	2.976373	0.85475	5.675735
50	H	-5.25319	4.205881	-1.03432
51	H	-3.9831	5.037424	-1.96773
52	H	-4.11042	5.332245	-0.23535
53	H	5.074392	-0.75438	-4.27616
54	H	5.971418	0.09411	-2.97896
55	H	5.065407	1.004979	-4.22488
56	H	4.981976	4.152328	0.985924
57	H	3.638505	5.171915	1.586569

58	H	4.038577	5.186275	-0.12825
59	H	4.195198	-5.07527	-0.22546
60	H	3.788701	-5.11417	1.487469
61	H	5.102058	-4.03937	0.916464
62	H	-5.03002	-0.97799	4.102518
63	H	-5.98916	-0.08614	2.88468
64	H	-5.03653	0.782382	4.125018
65	H	-5.01413	1.027338	-1.41182
66	H	-5.40488	-0.49056	-1.21815
67	H	-0.12292	0.034736	-4.67484

PAQMAY before hydrogenation

	Atom	X	Y	Z
1	O	4.706353	0.076174	-0.92228
2	C	4.212288	-1.19843	-1.26514
3	O	2.138328	1.420778	-1.92471
4	O	-1.101	-2.49037	-0.73105
5	O	0.800376	-1.38868	-1.08018
6	C	0.093563	-2.45097	-1.11248
7	C	0.763893	-3.70188	-1.66454
8	O	-3.13255	0.090349	-1.75645
9	C	-3.769	0.88785	-2.69325
10	O	-3.80223	-0.23925	1.346686
11	C	-5.13028	0.084031	1.633131
12	O	-0.22294	-0.58024	1.574052
13	O	1.95742	-0.47995	1.918478
14	C	0.804017	-0.88207	2.255716
15	C	0.686954	-1.76628	3.466665
16	O	0.717595	2.689441	0.766275
17	O	-0.84424	1.507682	-0.23251
18	C	-0.43909	2.615292	0.289721
19	C	-1.33753	3.811475	0.239198
20	O	4.003945	0.46044	0.261929
21	C	4.838127	0.11611	1.355106
22	Tb	1.462788	0.606962	-0.15943
23	Tb	-2.20039	-0.43926	0.011225
24	H	3.165212	-1.17082	-1.58501
25	H	4.34586	-1.91838	-0.4486

26	H	4.826009	-1.50902	-2.11557
27	H	1.013703	-3.52782	-2.71494
28	H	0.105817	-4.56567	-1.5787
29	H	1.699346	-3.87967	-1.12748
30	H	-3.21263	1.821435	-2.87691
31	H	-4.78487	1.165874	-2.37015
32	H	-3.86488	0.37309	-3.66166
33	H	-5.673	0.422853	0.73663
34	H	-5.19052	0.888408	2.382239
35	H	-5.67405	-0.78123	2.041388
36	H	-0.26955	-1.60962	3.968323
37	H	1.519898	-1.59091	4.147321
38	H	0.722739	-2.80925	3.133799
39	H	-2.35375	3.535637	0.530838
40	H	-1.37806	4.155642	-0.79911
41	H	-0.95239	4.608721	0.87337
42	H	4.26663	0.380658	2.245547
43	H	5.769109	0.684618	1.296957
44	H	5.039481	-0.95904	1.370071
45	H	2.717636	1.761898	-2.60203

PAQMAY after hydrogenation

	Atom	X	Y	Z
1	O	4.598195	0.191756	-0.89332
2	C	4.277247	-1.14532	-1.25657
3	O	2.515768	1.621541	-1.85654
4	O	-0.8471	-2.30342	-1.00974
5	O	1.260492	-1.52175	-1.01741
6	C	0.374433	-2.42399	-1.23071
7	C	0.948877	-3.7116	-1.7829
8	O	-3.26173	0.263482	-1.62438
9	C	-3.73965	0.752262	-2.81863
10	O	-3.81386	-0.44727	1.311998
11	C	-5.16358	-0.12317	1.473571
12	O	-0.17062	-0.98125	1.36112
13	O	1.748964	-0.18416	2.10601
14	C	0.742717	-0.9458	2.234776
15	C	0.667042	-1.87696	3.387929
16	O	0.781677	2.881656	-0.02699
17	O	-0.69317	1.576246	0.9676

18	C	-0.38921	2.684342	0.413668
19	C	-1.45265	3.68933	0.204589
20	O	3.907524	0.439202	0.339243
21	C	4.810417	0.146726	1.395144
22	Tb	1.235507	0.540767	-0.07395
23	Tb	-2.15036	-0.3997	-0.01899
24	H	3.211256	-1.29231	-1.43774
25	H	4.631105	-1.84706	-0.49416
26	H	4.844816	-1.31339	-2.17658
27	H	1.466017	-3.49831	-2.72248
28	H	0.171697	-4.45734	-1.95159
29	H	1.690173	-4.10166	-1.07978
30	H	-2.9384	1.197316	-3.43244
31	H	-4.50527	1.531194	-2.66512
32	H	-4.2085	-0.04007	-3.42461
33	H	-5.61793	0.216461	0.528016
34	H	-5.30066	0.680838	2.215195
35	H	-5.7462	-0.98858	1.827768
36	H	-0.35576	-1.92337	3.768038
37	H	1.358728	-1.58543	4.178237
38	H	0.927982	-2.88117	3.037522
39	H	-2.17007	3.67125	1.026632
40	H	-1.98883	3.405003	-0.70937
41	H	-1.03758	4.688056	0.066404
42	H	4.216447	0.285386	2.300501
43	H	5.658667	0.834471	1.363871
44	H	5.149248	-0.8917	1.342425
45	H	3.473309	1.475052	-1.79732
46	H	2.328616	2.567057	-1.80959
47	H	-0.45667	0.535554	-1.29908

ZIJSAO before hydrogenation

	Atom	X	Y	Z
1	O	-0.46296	1.567392	1.136348
2	O	0.527182	1.733667	-0.8286
3	C	-0.11716	3.767857	0.259509
4	C	0.006467	2.282856	0.187854
5	O	0.889135	-1.57553	1.707094

6	O	-2.19903	-1.2489	0.648292
7	O	-2.07128	-0.23048	-1.30007
8	C	-4.25403	-0.43331	-0.30702
9	C	-2.76992	-0.67192	-0.33021
10	O	2.39628	-0.22847	0.881232
11	O	1.888233	-1.14197	-1.07826
12	C	4.108045	-0.25405	-0.7684
13	C	2.716862	-0.59336	-0.30187
14	Tb	-0.0921	-0.38955	-0.02392
15	H	-1.09679	4.047157	-0.14259
16	H	0.651216	4.24647	-0.34814
17	H	-0.06899	4.106227	1.295377
18	H	-4.64832	-0.38304	-1.32252
19	H	-4.43127	0.53165	0.17928
20	H	-4.75633	-1.20658	0.275106
21	H	4.799378	-0.21109	0.072933
22	H	4.069772	0.729926	-1.24624
23	H	4.432832	-0.98394	-1.51151
24	H	1.152904	-1.15028	2.535582

ZIJSAO after hydrogenation

	Atom	X	Y	Z
1	O	-0.46581	1.587485	1.065786
2	O	0.450001	1.75209	-0.93585
3	C	0.099858	3.779191	0.282671
4	C	0.015933	2.297969	0.122752
5	O	0.159082	-1.37653	2.098282
6	O	-2.17729	-1.13984	0.635503
7	O	-2.02875	-0.2436	-1.37125
8	C	-4.19662	-0.25144	-0.33326
9	C	-2.72994	-0.5847	-0.36798
10	O	2.227782	-0.09954	0.831322
11	O	1.952792	-1.20438	-1.05942
12	C	4.175732	-0.44164	-0.5274
13	C	2.711818	-0.60596	-0.2221
14	Tb	-0.02442	-0.37909	-0.09913
15	H	0.065438	4.269929	-0.69054
16	H	1.059429	4.018707	0.753198
17	H	-0.69665	4.140651	0.93427
18	H	-4.60912	-0.24112	-1.34275

19	H	-4.30183	0.752846	0.090269
20	H	-4.73549	-0.95187	0.305863
21	H	4.747209	-0.33215	0.394749
22	H	4.293261	0.472879	-1.11809
23	H	4.542309	-1.27918	-1.12237
24	H	0.882487	-0.92551	2.551603
25	H	-0.65374	-1.27161	2.606382
26	H	-0.40899	-3.37861	-0.76217

c5dt03091a_c5dt03091a2 before hydrogenation

	Atom	X	Y	Z
1	O	0.318638	-1.64917	2.971075
2	O	0.296505	0.560042	3.34747
3	C	0.634336	-0.91017	5.213688
4	C	0.416684	-0.64116	3.740554
5	Er	-0.03854	1.634743	1.329247
6	O	-1.79147	3.047599	0.986364
7	O	-2.86381	2.189738	-0.77556
8	O	-0.11157	1.523123	-0.9816
9	H	-0.23557	2.349568	-1.47539
10	C	-2.67017	3.119241	0.059075
11	C	-3.60871	4.298583	-0.00095
12	Er	-0.0382	-2.01801	0.756418
13	O	-2.03943	-3.08936	0.451236
14	O	-3.06137	-1.80842	-1.06259
15	O	-1.38686	-0.1549	0.8617
16	H	-2.23057	-0.21791	1.340905
17	C	-3.05299	-2.78425	-0.26323
18	C	-4.34414	-3.55747	-0.10508
19	O	3.002387	-1.62623	-1.21438
20	O	1.954855	-3.03121	0.174148
21	C	4.142061	-3.62579	-0.65828
22	C	2.94936	-2.69577	-0.54854
23	O	1.924421	2.768877	1.113474
24	O	3.01297	1.874756	-0.62333
25	C	4.092838	3.630383	0.568182
26	C	2.934577	2.682702	0.339269
27	Er	1.719888	0.234468	-1.64163

28	O	1.942847	0.583704	-3.62451
29	O	-0.13621	-1.22767	-1.39641
30	H	-0.26382	-1.81708	-2.15845
31	Er	-2.11118	0.204927	-1.44586
32	O	1.303157	-0.13963	0.705779
33	H	2.112751	-0.22495	1.221706

c5dt03091a_c5dt03091a2 after hydrogenation

	Atom	X	Y	Z
1	O	-0.45569	-1.74606	2.950036
2	O	-0.11682	0.415097	3.426345
3	C	-0.06071	-1.17346	5.22374
4	C	-0.23168	-0.80039	3.766639
5	Er	0.079835	1.577828	1.423759
6	O	-1.41733	3.266262	1.031637
7	O	-2.49416	2.575193	-0.79826
8	O	0.255226	1.558765	-0.81253
9	H	0.467696	2.310107	-1.39251
10	C	-2.22233	3.458364	0.055187
11	C	-2.87522	4.824787	-0.10802
12	Er	-0.48237	-2.02464	0.680101
13	O	-2.60267	-2.69003	0.154288
14	O	-3.33771	-1.05145	-1.17579
15	O	-1.49715	0.070262	0.724398
16	H	-2.37796	0.080295	1.115
17	C	-3.5165	-2.12139	-0.54505
18	C	-4.88349	-2.78901	-0.62531
19	O	2.60345	-2.18588	-1.1508
20	O	1.339654	-3.38193	0.25085
21	C	3.488983	-4.26914	-0.4311
22	C	2.390791	-3.21742	-0.45569
23	O	2.207197	2.359597	1.460499
24	O	3.197358	1.534334	-0.37058
25	C	4.521997	2.853028	1.107411
26	C	3.214544	2.219487	0.686438
27	Er	1.859936	-0.03682	-1.44763
28	O	3.368343	0.608884	-3.13842
29	O	-0.25436	-1.22773	-1.4045
30	H	-0.19314	-1.73026	-2.23293

31	Er	-1.73921	0.588587	-1.7323
32	O	1.148288	-0.40104	0.834241
33	H	1.923167	-0.63294	1.360784
34	H	-0.82524	-1.8972	5.512327
35	H	-0.09856	-0.29132	5.862093
36	H	0.912448	-1.66146	5.340629
37	H	-2.80157	5.409372	0.808842
38	H	-3.9145	4.696555	-0.41412
39	H	-2.35607	5.351527	-0.91486
40	H	-4.84049	-3.55925	-1.40215
41	H	-5.64054	-2.0568	-0.90404
42	H	-5.12762	-3.27711	0.319681
43	H	3.126885	-5.21238	-0.02222
44	H	4.302341	-3.8917	0.197816
45	H	3.885251	-4.40352	-1.43851
46	H	5.188526	2.961357	0.252602
47	H	4.997575	2.192131	1.841248
48	H	4.344681	3.814639	1.591706
49	H	3.817141	1.45646	-2.97602
50	H	-2.75714	0.864792	-3.45903
51	H	2.622389	0.796918	-3.77099

Text S1. Frequency Analysis

The frequency calculations of MOF structures were conducted using the PBE0 functional. For metal elements, the pseudopotentials of Stuttgart group was employed, while for main-group elements (C, H, O), the 6-31G(d) basis set was used. All DFT calculations incorporated the Grimme's D3 dispersion correction to account for weak interactions. The Gaussian convergence criteria were applied as maximum force < 0.00045 a.u., RMS force < 0.0003 a.u., maximum displacement < 0.0018 a.u.

All geometry-optimized structures were subjected to vibrational frequency calculations at the same level of theory to confirm their nature as true minima (no imaginary frequencies). The absence of imaginary frequencies verified that each optimized structure corresponds to a local minimum on the potential energy surface.
